# Microvasculature recovery in lamina cribrosa and peripapillary sclera after glaucoma surgery and its impact on visual field progression

**DOI:** 10.1038/s41598-025-08205-w

**Published:** 2025-07-11

**Authors:** Hee Jong Shin, Hee Kyung Ryu, Seong Ah Kim, Younhea Jung, Chan Kee Park, Hae-Young Lopilly Park

**Affiliations:** 1 Department of Ophthalmology, Seoul St. Mary’s Hospital, College of Medicine, The Catholic University of Korea, Seoul, South Korea; 2 Department of Ophthalmology, Yeouido St. Mary’s Hospital, College of Medicine, The Catholic University of Korea, Seoul, South Korea; 3 Department of Ophthalmology and Visual Science, Seoul St. Mary’s Hospital, College of Medicine, The Catholic University of Korea, 505 Banpo-dong, Seocho-ku, Seoul, 137-701 South Korea

**Keywords:** Glaucoma surgery, Microvasculature recovery, Lamina cribrosa, Peripapillary sclera, Ocular hypertension, Optic nerve diseases

## Abstract

This study investigates vessel density (VD) changes in the lamina cribrosa (LC) and peripapillary sclera (PPS) after glaucoma surgery and their association with visual field (VF) progression. Primary open-angle glaucoma patients undergoing surgery for uncontrolled intraocular pressure (IOP) at Seoul St. Mary’s Hospital were included. Optical coherence tomography angiography (OCT-A) assessed VD changes in the LC and PPS one month post-surgery. VF progression was evaluated using mean deviation (MD) values from serial VF tests over six months. Of 80 enrolled eyes, 74 were analyzed. Laminar VD recovery occurred in 12 eyes (16.2%), with a 21.92% ± 7.37% increase, linked to shorter axial length (P = 0.005), thinner corneal thickness (P = 0.016), and less PPS VD change (P < 0.001). PPS VD recovery occurred in 14 eyes (18.9%), with an 18.50% ± 7.28% increase, associated with younger age (P = 0.043), longer axial length (P = 0.010), and lower preoperative PPS VD (P < 0.001). Multivariate analysis showed that both laminar and PPS VD recovery significantly reduced VF progression risk (P < 0.001). VD recovery, particularly in the LC, predicts better glaucoma outcomes. The distinct responses of LC and PPS to IOP reduction highlight the need to consider individual anatomical factors in glaucoma management.

## Introduction

Glaucoma is a progressive disease that involves the optic nerve, and structural changes occur within the lamina cribrosa (LC) and peripapillary sclera (PPS)^1^. The PPS plays a significant role in the biomechanics and structural integrity of the optic nerve head (ONH). Studies have highlighted the importance of understanding the anatomy and histomorphometry of both the LC and PPS in relation to the ONH, particularly in conditions such as glaucoma^2,3^. Researchers have investigated the architecture of the PPS and its contribution to the biomechanics of the ONH, emphasizing its relevance to optic disc boundaries and ONH structure^4,5^. 

As glaucoma progresses, structural changes in the ONH occur, and a decrease in the microvasculature of the LC and PPS is observed^6^. However, other than the loss of microvasculature due to glaucoma progression, many factors contribute to changes in the ONH microvasculature, of which intraocular pressure (IOP) is an important one. Several studies have reported that blood flow at the ONH is restored after IOP reduction from trabeculectomy^7–12^. Our previous study also showed that a postoperative increase in deep vessel density (VD) of the laminar region is beneficial for visual field (VF) progression^13^. Additionally the tissue properties of the ONH may influence changes in the microvasculature. A study has shown that the degree of peripapillary VD recovery after surgery was reduced in advanced glaucoma, even with the same degree of IOP reduction^14^. However, many contributing factors other than IOP and the tissue properties of the ONH may influence the changes in the ONH microvasculature, and reports are controversial^15–17^.

The development of glaucoma depends on the responses of the LC and PPS and how these two structures are matched or mismatched in their combined response to IOP-induced stress^18^. Therefore, to analyze the clinical significance of the ONH microvasculature, it is necessary to observe the structural changes in both the LC and PPS. In addition, since changes in the PPS are more frequently observed in myopia^19^, we hypothesized that the significance of these changes might differ between glaucoma with or without myopia.

In this study, we investigated the characteristics associated with changes in VD by dividing the area into the LC and PPS regions after IOP reduction during glaucoma surgery. Additionally, we compared VF progression after glaucoma surgery in terms of the location of VD recovery to determine its clinical significance.

## Results

Eighty eyes of 80 patients who underwent glaucoma surgery met the inclusion criteria and underwent serial OCT-A imaging. Of these, six (7.5%) were excluded from further analysis because the OCT-A images were of poor quality or contained motion artifacts. Interobserver agreement in terms of VD measurement was excellent (ICC = 0.916; 95% CI 0.877–0.953; P < 0.001). In addition, the 95% Bland–Altman limits of agreement between repeated measurements were − 1.47% to 1.04% for the intradisc VD and − 1.52% to 1.17% for the PPA area in the deep vascular layer, indicating acceptable test–retest variability.

Baseline patient characteristics are listed in Table 1. The mean patient age was 51.89 ± 19.47 years, and 31 (41.9%) patients were female. All patients were taking the maximum-tolerated dose of glaucoma medication. The mean spherical equivalent refractive error was − 3.29 ± 4.08 D, and the mean axial length was 25.45 ± 1.89 mm. The preoperative MD of the VF was − 3.22 ± 4.15 dB. The total follow-up period was 0.65 ± 0.02 years after surgery. The mean preoperative IOP of 31.30 ± 7.15 mmHg was reduced significantly to 14.63 ± 3.28 mm Hg at one month (P < 0.001) after glaucoma surgery.

**Table 1:** Preoperative characteristics and parameters of OCT-A of 74 eyes of 74 glaucoma patients who undergone glaucoma surgery.

Variables	Description
Age, year	51.89 ± 19.47
Female, n (%)	31 (41.9%)
Spherical equivalent, diopters	− 3.29 ± 4.08
Axial length, mm	25.45 ± 1.89
Central corneal thickness, μm	543.71 ± 37.16
Preoperative IOP, mmHg	31.30 ± 7.15
Postoperative IOP, mmHg	14.63 ± 3.28
Preoperative average pRNFL thickness, μm	65.26 ± 12.46
Preoperative average mGC/IPL thickness, μm	61.01 ± 12.35
Preoperative MD of VF, dB	− 3.22 ± 4.15
Follow-up period, year	0.65 ± 0.02

IOP, intraocular pressure; pRNFL, peripapillary retinal nerve fiber layer; mGC/IPL, macular ganglion cell-inner plexiform layer; VF, visual field; MD, mean deviation; PSD, pattern standard deviation; dB, decibel.

Data are mean ± standard deviation unless otherwise indicated.

Of 74 eyes, 12 (16.2%) showed a laminar VD change one month after glaucoma surgery. The laminar VD change and no change groups had increase in the laminar VD 21.92% ± 7.37% and 1.77% ± 4.49%, respectively (P < 0.001; Table2). Shorter axial length (P = 0.005), thinner central corneal thickness (P = 0.016), and less change in the PPS VD (P < 0.001) were significantly associated with glaucomatous eyes with laminar VD changes after glaucoma surgery. When analyzing PPS VD, 14 (18.9%) of the 74 eyes showed a significant change. The PPS VD change and no change groups showed an increase in the PPS VD 18.50% ± 7.28% and 0.87% ± 2.15%, respectively (P < 0.001; Table3). Younger age (P = 0.043), lower spherical equivalent (P = 0.044), longer axial length (P = 0.010), and lower preoperative PPS VD (P < 0.001) were significantly associated with changes in the PPS VD eyes after glaucoma surgery. Interestingly, less change in the laminar VD (P = 0.065) showed borderline significance with changes in the PPS VD.

**Table 2:** Comparison between glaucoma patients with and without laminar VD change after glaucoma surgery.

Variables	Laminar VD change (+) (n = 12)	Laminar VD change (−) (n = 62)	P value
Age, y	53.33 ± 17.13	51.61 ± 20.02	0.761*
Female, n (%)	4 (33.3%)	27 (43.5%)	0.373^†^
Diagnosis			0.398^†^
POAG, n (%)	8 (66.7%)	43 (69.4%)	
JOAG, n (%)	0	6 (9.7%)	
Others, n (%)	4 (33.3%)	13 (21.0%)	
Spherical equivalent, diopters	− 1.91 ± 2.65	− 3.51 ± 4.31	0.174*
Axial length, mm	24.36 ± 1.10	25.67 ± 1.94	0.005*
Central corneal thickness, μm	521.44 ± 24.27	547.42 ± 37.78	0.016*
Preoperative IOP, mmHg	32.29 ± 5.68	31.11 ± 7.42	0.541*
Postoperative IOP, mmHg	13.24 ± 3.23	14.90 ± 3.25	0.124*
Change of IOP, %	− 70.40 ± 16.46	− 55.69 ± 25.35	0.101*
Average pRNFL thickness, μm	64.77 ± 13.04	65.33 ± 12.49	0.906*
Average mGC/IPL thickness, μm	62.55 ± 9.15	60.74 ± 12.86	0.616*
MD of VF, dB	− 12.71 ± 8.15	− 15.19 ± 9.42	0.470*
PSD of VF, dB	8.22 ± 4.45	9.17 ± 8.83	0.355*
Preoperative laminar VD, %	11.58 ± 5.68	13.72 ± 8.40	0.286*
Change of laminar VD, %	21.92 ± 7.37	1.77 ± 4.49	< 0.001*
Preoperative PPS VD, %	13.75 ± 8.77	9.32 ± 7.44	0.123
Change of PPS VD, %	0.33 ± 0.65	5.46 ± 8.54	< 0.001*
Follow-up period, y	0.66 ± 0.02	0.65 ± 0.02	0.367*

IOP, intraocular pressure; pRNFL, peripapillary retinal nerve fiber layer; mGC/IPL, macular ganglion cell-inner plexiform layer; VF, visual field; MD, mean deviation; PSD, pattern standard deviation; dB, decibel; PPS, peripapillary sclera; VD, vessel density; FAZ, foveal avascular zone.

Data are mean ± standard deviation unless otherwise indicated.

*Student’s t-test.

^†^Chi-square test.

Factors with statistical significance are shown in bold.

**Table 3:** Comparison between glaucoma patients with and without PPS VD change after glaucoma surgery.

Variables	PPS VD change +) (n = 14)	PPS VD change (−) (n = 60)	P value
Age, y	41.79 ± 19.48	54.25 ± 18.866	0.043*
Female, n (%)	7 (50.0%)	24 (40.0%)	0.348^†^
Diagnosis			0.231^†^
POAG, n (%)	7 (50.0%)	44 (73.3%)	
JOAG, n (%)	2 (14.3%)	4 (6.7%)	
Others, n (%)	5 (35.7%)	12 (20.0%)	
Spherical equivalent, diopters	− 5.90 ± 5.01	− 2.67 ± 3.61	0.044*
Axial length, mm	26.67 ± 1.69	25.15 ± 1.82	0.010*
Central corneal thickness, μm	548.25 ± 33.29	542.64 ± 38.23	0.617*
Preoperative IOP, mmHg	30.50 ± 6.92	31.49 ± 7.24	0.638*
Postoperative IOP, mmHg	14.88 ± 14.57	14.57 ± 3.53	0.663*
Change of IOP, %	50.24 ± 31.07	60.20 ± 22.84	0.450*
Average pRNFL thickness, μm	64.64 ± 12.49	65.42 ± 12.57	0.837*
Average mGC/IPL thickness, μm	56.81 ± 7.92	61.91 ± 12.99	0.103*
MD of VF, dB	− 13.56 ± 9.04	− 15.112 ± 9.32	0.572*
PSD of VF, dB	7.73 ± 4.68	9.34 ± 8.96	0.356*
Preoperative laminar VD, %	11.64 ± 9.51	13.78 ± 7.68	0.444*
Change of laminar VD, %	1.71 ± 6.59	5.81 ± 9.34	0.065*
Preoperative PPS VD, %	3.42 ± 5.16	12.11 ± 7.21	< 0.001*
Change of PPS VD, %	18.50 ± 7.28	0.87 ± 2.15	< 0.001*
Follow-up period, y	0.65 ± 0.03	0.66 ± 0.15	0.425*

Table 4 shows a comparison of the pattern of VD change in the two areas according to VF progression. After glaucoma surgery, 12 eyes exhibited an increase in laminar VD in the non-progression group, compared with no eyes in the progression group (P = 0.012). Interestingly, PPS VD recovery was observed in a higher proportion of eyes in the VF progression group (46.7%) compared to the non-progression group (11.9%) (P = 0.006). Although counterintuitive, this finding emphasizes the need for multivariate analysis, which revealed that PPS VD recovery was significantly associated with a reduced risk of VF progression only when adjusted for additional clinical factors (P < 0.001, Table 8). There was a significant difference in the location of VD change between the progression and non-progression groups (P = 0.005). Twelve eyes with a laminar VD increase and 7 eyes with a PPS VD increase were observed in the non-progression group.

**Table 4:** Distribution of the patients showing significant changes in the parameters of OCT-A at postoperative 1 month after glaucoma surgery.

OCT angiography parameters	Progression after glaucoma surgery		Total	P value*
	Progressor	Non-progressor		
Laminar VD				
No change	21 (33.9%)	41 (66.1%)	62	0.012
Increase	0	12 (100%)	12	
PPS VD change				
No change	8 (13.3%)	52 (86.7%)	60	0.006
Increase	7 (50.0%)	7 (50.0%)	14	
Increase laminar VD	0	12 (100%)	12	0.005
Increase PPS VD	7 (50.0%)	7 (50.0%)	14	

PPS, peripapillary sclera; VD, vessel density.

*Chi-square test.

We performed linear regression analysis to identify the factors associated with preoperative PPS VD (Table 5). Older age (P = 0.057), shorter axial length (P = 0.003), and higher preoperative laminar VD in the deep vascular layer (P = 0.014) were significantly associated with preoperative PPS VD in the univariate analysis. Among these factors, shorter axial length (P = 0.050) and higher preoperative laminar VD (P = 0.007) were significantly associated with preoperative PPS VD in the multivariate analysis.

**Table 5:** Factors associated with the preoperative PPS VD in glaucoma patients who undergone glaucoma surgery.

Variables	Univariate			Multivariate		
	Beta	95% CI	*P* value	Beta	95% CI	*P* value
Age, per 1 y older	0.087	− 0.003 to 0.177	0.057	0.087	− 0.011 to 0.185	0.082
Axial length, per 1 mm larger	− 1.516	− 2.482 to − 0.549	**0.003**	− 0.992	− 2.005 to − 0.020	**0.050**
Central corneal thickness, per 1 μm thicker	− 0.009	− 0.060 to 0.042	0.724			
Preoperative av pRNFLT, per 1 μm thicker	− 0.063	− 0.213 to 0.087	0.403			
Preoperative av mGC/IPLT, per 1 μm thicker	0.016	− 0.145 to 0.178	0.841			
Preoperative MD of VF, per 1 dB higher	− 0.057	− 0.254 to 0.140	0.656			
Preoperative PSD of VF, per 1 dB higher	0.041	− 0.179 to 0.261	0.710			
Preoperative IOP, per 1 mmHg higher	− 0.100	− 0.351 to 0.151	0.429			
Postoperative IOP, per 1 mmHg higher	− 0.123	− 0.670 to 0.424	0.656			
Preoperative laminar VD, per 1% higher	0.272	0.057 to 0.486	**0.014**	0.299	0.083 to 0.515	**0.007**

CI, confidence interval; av, average; pRNFLT, peripapillary retinal nerve fiber layer thickness; mGC/IPLT, macular ganglion cell-inner plexiform layer thickness; IOP, intraocular pressure; VF, visual field; MD, mean deviation; PSD, pattern standard deviation; dB, decibel; PPS, peripapillary sclera; VD, vessel density.

Factors with *P* < 0.2 in univariate analysis were included in multivariate analysis.

Factors with statistical significance are shown in bold.

We subsequently analyzed the factors affecting the changes in PPS VD and laminar VD were analyzed using linear regression analysis (Tables 6 and 7, respectively). Younger age (P = 0.026) and longer axial length (P = 0.014) were significant factors affecting changes in PPS VD in the univariate analysis, and only longer axial length (P = 0.001) was found to significantly influence PPS VD change in the multivariate analysis. In contrast, shorter axial length (P = 0.019) was found to be significant in the univariate analysis related to changes in laminar VD but was not shown to be significantly associated in the multivariate analysis (P = 0.211).

**Table 6:** Factors associated with the change of PPS VD in glaucoma patients who undergone glaucoma surgery.

Variables	Univariate			Multivariate		
	Beta	95% CI	*P* value	Beta	95% CI	*P* value
Age, per 1 y older	− 0.034	0.938 to 0.996	**0.026**	− 0.023	0.943 to 1.013	0.206
Female gender	− 0.405	0.207 to 2.144	0.496			
Axial length, per 1 mm larger	0.425	1.090 to 2.147	**0.014**	0.485	1.014 to 2.151	**0.001**
Central corneal thickness, per 1 μm thicker	0.006	0.990 to 1.023	0.437			
Preoperative av pRNFLT, per 1 μm thicker	0.009	0.965 to 1.055	0.699			
Preoperative av mGC/IPLT, per 1 μm thicker	− 0.021	0.934 to 1.027	0.396			
Preoperative MD of VF, per 1 dB higher	− 0.010	0.932 to 1.052	0.755			
Preoperative PSD of VF, per 1 dB higher	− 0.036	0.865 to 1.076	0.516			
Preoperative IOP, per 1 mmHg higher	0.008	0.933 to 1.090	0.838			
Postoperative IOP, per 1 mmHg higher	0.080	0.917 to 1.280	0.346			
Preoperative laminar VD, per 1% higher	− 0.017	0.915 to 1.057	0.645			
Change of laminar VD, per 1% higher	− 0.061	0.866 to 1.023	0.255			
Image quality score	0.085	0.790 to 1.300	0.620			
Follow-up period, year	− 0.015	0.916 to 1.004	0.729			

CI, confidence interval; av, average; pRNFLT, peripapillary retinal nerve fiber layer thickness; mGC/IPLT, macular ganglion cell-inner plexiform layer thickness; IOP, intraocular pressure; VF, visual field; MD, mean deviation; PSD, pattern standard deviation; dB, decibel; PPS, peripapillary sclera; VD, vessel density.

Factors with *P* < 0.2 in univariate analysis were included in multivariate analysis.

Factors with statistical significance are shown in bold.

**Table 7:** Factors associated with the changes in laminar VD in glaucoma patients who undergone glaucoma surgery.

Variables	Univariate			Multivariate		
	Beta	95% CI	*P* value	Beta	95% CI	*P* value
Age, per 1 y older	0.005	0.973 to 1.038	0.778			
Female gender	0.434	0.420 to 5.667	0.514			
Axial length, per 1 mm larger	− 0.166	− 0.305 to − 0.028	**0.019**	− 0.062	0.496 to 1.784	0.850
Central corneal thickness, per 1 μm thicker	− 0.022	0.957 to 1.001	0.058	− 0.017	0.957 to 1.010	0.211
Preoperative av pRNFLT, per 1 μm thicker	− 0.004	0.941 to 1.055	0.899			
Preoperative av mGC/IPLT, per 1 μm thicker	0.017	− 0.050 to 0.015	0.290			
Preoperative MD of VF, per 1 dB higher	− 0.028	0.906 to 1.043	0.427			
Preoperative PSD of VF, per 1 dB higher	0.013	0.952 to 1.078	0.681			
Preoperative IOP, per 1 mmHg higher	0.023	0.939 to 1.116	0.102	0.037	0.912 to 1.180	0.576
Postoperative IOP, per 1 mmHg higher	− 0.177	0.574 to 1.041	0.111	− 0.093	0.686 to 1.211	0.521
Preoperative PPS VD, per 1% higher	0.066	0.985 to 1.160	0.111	0.068	0.950 to 1.206	0.266
Changes in PPS VD, per 1% higher	− 0.271	0.513 to 1.134	0.181	− 0.199	0.461 to 1.459	0.500
Image quality score	0.007	0.833 to 1.004	0.256			
Follow-up period, year	− 0.005	0.923 to 1.006	0.620			

CI, confidence interval; av, average; pRNFLT, peripapillary retinal nerve fiber layer thickness; mGC/IPLT, macular ganglion cell-inner plexiform layer thickness; IOP, intraocular pressure; VF, visual field; MD, mean deviation; PSD, pattern standard deviation; dB, decibel; PPS, peripapillary sclera; VD, vessel density.

Factors with *P* < 0.2 in univariate analysis were included in multivariate analysis.

Factors with statistical significance are shown in bold.

To further explore the factors influencing VF progression in patients undergoing glaucoma surgery, we performed an additional analysis summarized in Table 8. In the univariate analysis, greater changes in both laminar and PPS VD were significantly associated with less VF deterioration, as indicated by more positive MD slopes (both P = 0.001). In the multivariate analysis, this association remained significant (P < 0.001 for both), suggesting that recovery of VD in either region contributed to a slower rate of VF progression.

**Table 8:** Factors associated with the VF progression in glaucoma patients who undergone glaucoma surgery.

Variables	Univariate			Multivariate		
	Beta	95% CI	*P* value	Beta	95% CI	*P* value
Age, per 1 y older	0.985	0.961 to 1.010	0.244			
Female gender	0.878	0.3331 to 2.324	0.793			
Axial length, per 1 mm larger	1.015	0.775 to 1.329	0.914			
Central corneal thickness, per 1 μm thicker	0.993	0.978 to 1.008	0.328			
Preoperative av pRNFLT, per 1 μm thicker	0.988	0.947 to 1.031	0.575			
Preoperative av mGC/IPLT, per 1 μm thicker	0.982	0.940 to 1.026	0.615			
Preoperative MD of VF, per 1 dB higher	0.986	0.935 to 1.041	0.427			
Preoperative IOP, per 1 mmHg higher	1.093	0.927 to 1.163	0.833			
Postoperative IOP, per 1 mmHg higher	1.911	0.779 to 1.266	0.247			
Preoperative Laminar VD, per 1% higher	0.961	0.900 to 1.026	0.230			
Changes in Laminar VD, per 1% higher	0.966	0.944 to 0.989	**0.001**	0.970	0.955 to 0.983	** < 0.001**
Preoperative PPS VD, per 1% higher	0.948	0.885 to 1.014	0.120	1.013	0.896 to 1.146	0.839
Changes in PPS VD, per 1% higher	0.964	0.951 to 0.823	**0.001**	0.973	0.960 to 0.987	** < 0.001**
Image quality score	0.978	0.930 to 1.001	0.860			
Follow-up period, year	1.005	0.952 to 1.054	0.762			

CI, confidence interval; av, average; pRNFLT, peripapillary retinal nerve fiber layer thickness; mGC/IPLT, macular ganglion cell-inner plexiform layer thickness; IOP, intraocular pressure; VF, visual field; MD, mean deviation; PSD, pattern standard deviation; dB, decibel; PPS, peripapillary sclera; VD, vessel density.

Factors with *P* < 0.2 in univariate analysis were included in multivariate analysis.

Factors with statistical significance are shown in bold.

**Fig. 1:**
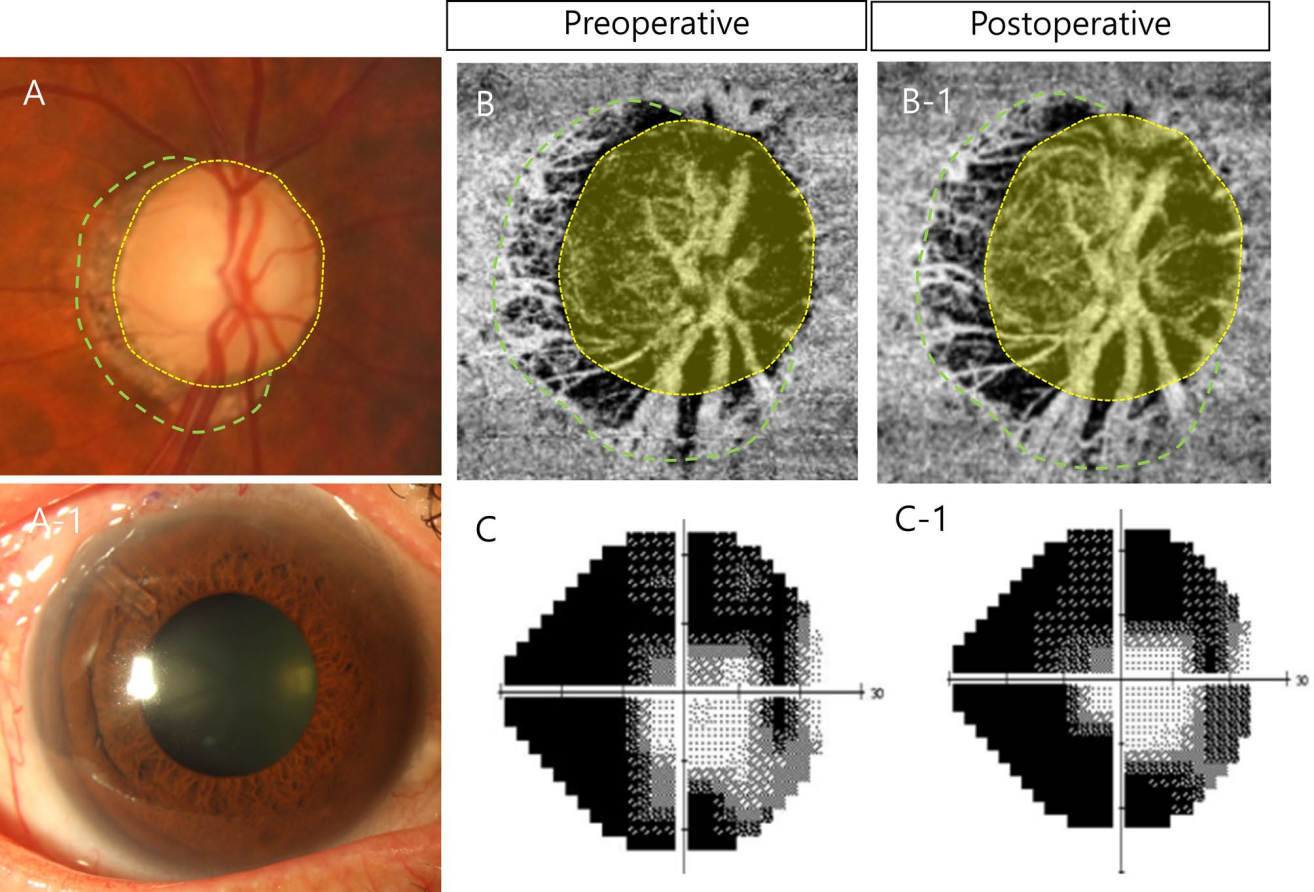
A 64-year-old man with glaucoma had uncontrolled IOP under the maximum tolerated medical treatment and underwent implantation of an Ahmed glaucoma drainage device (**A** and **1A–1**). This patient exhibited an increase in laminar VD on the deep vascular map at one month after surgery (**1B-1**, yellow shaded area) compared with the preoperative image (**B**). There was no change in the PPA area in the deep vascular layer (**1B-1**, area between the green dashed line and yellow dotted line). The patient did not exhibit any VF change after glaucoma surgery (**1C** and **1C-1**).

A representative case is shown in Fig. 1. A 64-year-old man with glaucoma had uncontrolled IOP under the maximum tolerated medical treatment and underwent implantation of an Ahmed glaucoma drainage device (Fig. 1A and A-1). This patient exhibited an increase in laminar VD on the deep vascular map at one month after surgery (1B-1, yellow shaded area) compared with the preoperative image (B). There was no change in the PPA area in the deep vascular layer (1B-1, area between the green dashed line and yellow dotted line). The patient did not exhibit any VF change after glaucoma surgery (1C and 1C-1).

**Fig. 2:**
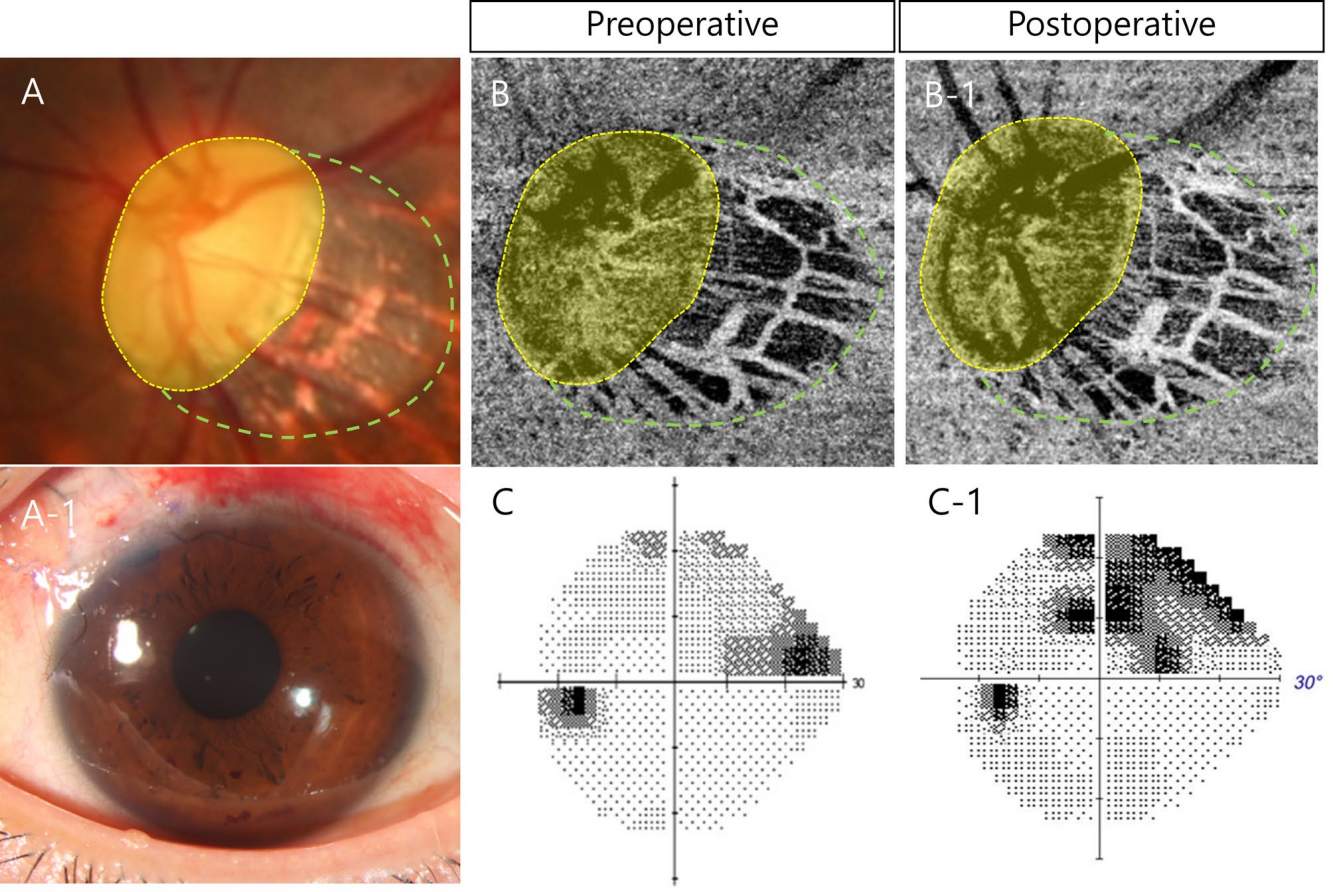
A 28-year-old woman with glaucoma underwent trabeculectomy (**A** and **A-1**) but unlike the first case, did not exhibit any changes in laminar VD after glaucoma surgery (**B** and **B-1**). There was a significant increase in VD in the PPA area, especially in the inferior part of the deep vascular layer (**B-1**, between the green dashed line and yellow dotted line). This patient showed VF progression with well-controlled IOP after glaucoma surgery (**C** and **C-1**).

Another representative case is shown in Fig. 2. A 28-year-old woman with glaucoma underwent trabeculectomy (Fig. 2A and A-1 but unlike the first case, did not exhibit any changes in laminar VD after glaucoma surgery (Fig. 2B and B-1). There was a significant increase in VD in the PPA area, especially in the inferior part of the deep vascular layer (2B-1, between the green dashed line and yellow dotted line). This patient showed VF progression with well-controlled IOP after glaucoma surgery (Fig. 2C and C-1).

**Fig. 3:**
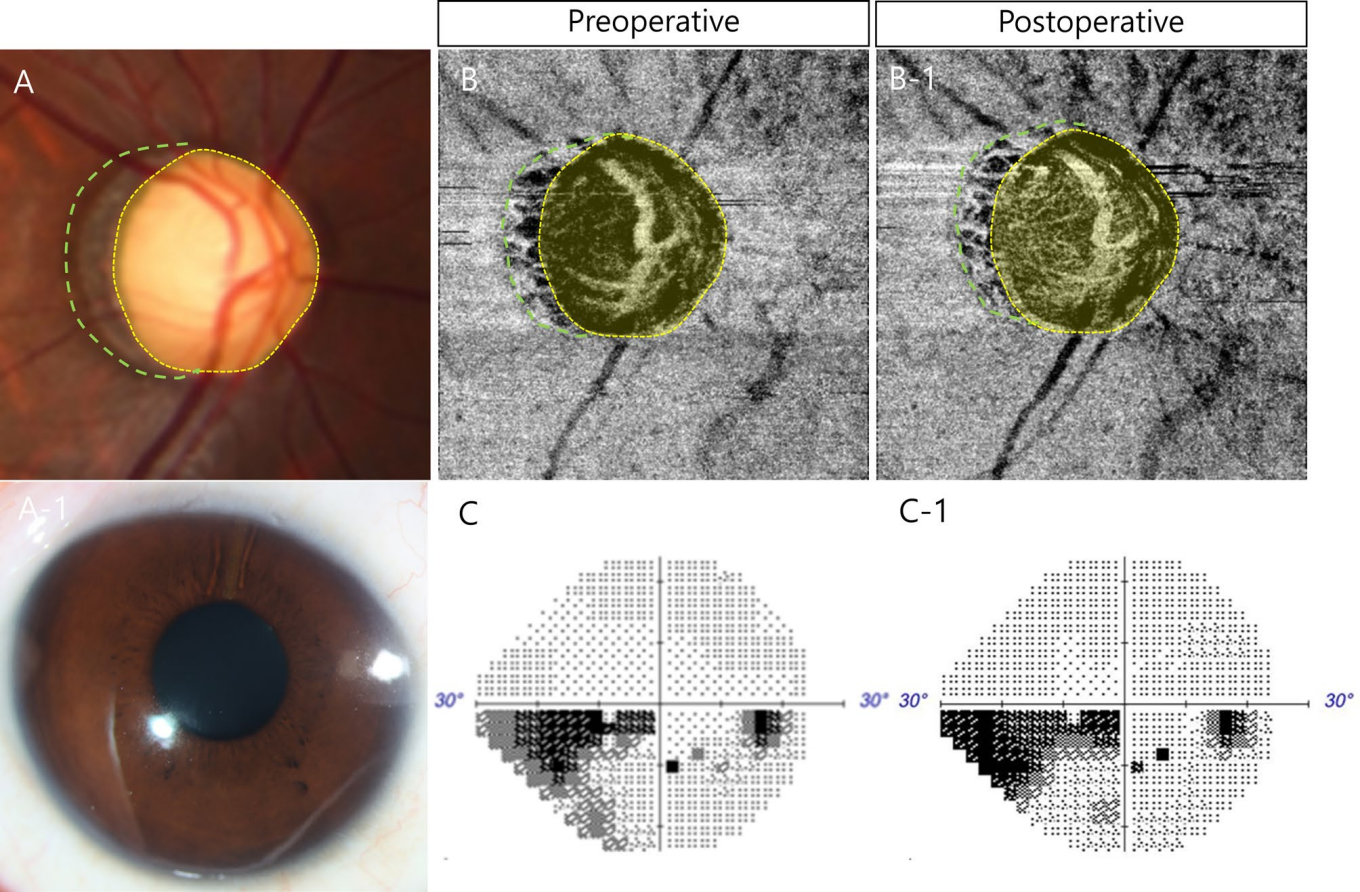
A 21-year-old woman with glaucoma who exhibited uncontrolled IOP under the maximum tolerated medical treatment (**A** and **A-1**). The patient underwent XEN glaucoma stent implantation. The VD inside the disc and PPA areas recovered after glaucoma surgery (**B** and **B-1**). VF progression was negligible after glaucoma surgery (**C** and **C-1**).

Figure 3 shows a representative case of a 21-year-old woman with glaucoma who exhibited uncontrolled IOP under the maximum tolerated medical treatment (Fig. 3A and A-1). The patient underwent XEN glaucoma stent implantation. The VD inside the disc and PPA areas recovered after glaucoma surgery (Fig. 3B and B-1). VF progression was negligible after glaucoma surgery (Fig. 3C and C-1).

## Discussion

We observed VD changes in the deep vascular layer of the lamina and PPS up to one month after glaucoma surgery. In this study, an increase in the deep VD in the intradisc area where the LC is located was evident in 12 eyes (16.2%), and an increase in the PPA area was found in 14 eyes (18.9%). The shorter the axial length and the thinner central corneal thickness were associated with grater significant recovery of VD at the laminar region. In addition, a smaller VD change in the PPA area was significantly associated with greater laminar VD recovery. Conversely, in the case of PPS VD recovery, more VD changes were found in myopic eyes. We may assume from these findings that factors affecting VD changes are different according to the ONH region.

Recovery of the VD means the capillaries compressed by high IOP in the connective tissue are observed again when the IOP decreases after glaucoma surgery because the strain applied to the area decreases. The fact that VD in a specific area does not recover is that, first, the strain may be applied to other parts of the ONH, and not enough strain is applied to compress the blood vessels in the area being examined. Second, the elasticity of the capillary wall has already decreased owing to changes in connective tissue composition around the vessel. The latter may indicate permanent tissue changes accompanying collapsed vessels within the tissue, which further exacerbate ischemic insults and result in VF progression.

The LC, a sieve-like structure within the ONH, is primarily supplied by the posterior ciliary arteries (PCAs) and branches of the ophthalmic artery (OA)^20^. Within the ONH, branches of the OA, particularly the short posterior ciliary arteries (SPCAs), provide the main vascular supply to the LC. These arteries penetrate the sclera around the ONH and supply neural and connective tissue elements to the LC. Additionally, the central retinal artery, which enters the eye through the optic nerve, contributes to the vascular supply of the ONH. The recovery of the laminar VD can be attributed to one of two possibilities. First, the LC itself may have been significantly compressed by the elevated IOP and subsequently returned to normal. Second, the PPS might have been compressed more than the LC, allowing the SPCAs entering the LC from the sclera to recover after glaucoma surgery. However, in the group in which laminar VD recovered, PPS VD hardly recovered and showed a significant negative correlation. From this, it is reasonable to assume that the strain from the elevated IOP was mainly concentrated on the LC of the ONHs, causing the capillaries within the LC to be compressed and then recovered. Among the associated factors to laminar VD recovery, central corneal thickness was one of the significant factor. Since thinner central corneal thickness may represent thinner or weak ONH structures, this means the stress from elevated IOP is applied to the ONH and easily compressing the microvasculature at the LC in eyes with thinner central corneal thickness supporting the above assumption. Recent findings also support the idea that structural alterations in the LC, such as reduced thickness or altered curvature, are closely associated with deep microvasculature dropout and higher IOP, emphasizing the importance of localized LC integrity in glaucomatous damage^21^.

The PPS and its relationship with the LC have been studied in the context of their role in protecting the ONH and influencing glaucoma pathophysiology. Burgoyne et al. illustrated how the mechanical environment of the ONH, including the interaction between the PPS and LC, can influence the susceptibility to glaucomatous damage^22^. Collagen fibers organized circumferentially and radially around the optic canal in the PPS are thought to provide biomechanical support to the sensitive tissues within the ONH^23^. The PPS provides a supportive framework that helps mitigate the transference of mechanical stress to the LC. Several studies have demonstrated histological and biomechanical differences between the sclera of myopic eyes and non-myopic eyes^24,25^. These differences include decreased collagen content, alterations in collagen fibril organization, and changes in the composition of proteoglycans, which are key components of the extracellular matrix in the sclera. As the degree of myopia increases, the PPS loses its protective role over the LC. Consequently, the LC is likely to have already undergone deformation due to chronically elevated IOP. As a result, it is believed that strain is mainly applied to structurally weak PPS. It is also possible that the degree of recovery of the PPS VD is large in myopic eyes because the relatively thinner sclera in myopia is more easily changed by an increase in IOP. In this study, greater significant VD recovery was observed in eyes with lower preoperative PPS VD, because the vessels were already compressed by the increase in IOP and this may represent easily deformable PPS in myopic eyes.

Our analysis further revealed that both laminar VD and PPS VD recovery were independently significant factors associated with reduced VF progression, as demonstrated in the multivariate analysis (Table 8). This underscores the importance of vascular recovery in both regions for determining postoperative prognosis. Interestingly, while both areas showed a significant association with VF outcomes, laminar VD recovery had a stronger correlation. This aligns with the hypothesis that the laminar region, being structurally and functionally critical for optic nerve head (ONH) integrity, plays a central role in glaucoma progression. The PPS, while also contributing to overall structural support and vascular supply, may have a more indirect influence on VF outcomes. This is in line with previous longitudinal data showing that reduction in optic disc microvasculature is significantly associated with visual field progression, even after adjusting for structural parameters such as RNFL thinning^26^.

These findings suggest that vascular recovery in the PPS may still provide a protective effect on the LC, potentially mitigating ischemic damage and strain transfer to the laminar region. However, the fact that PPS VD recovery was observed in some eyes where laminar VD recovery was absent indicates that localized remodeling or damage to the LC may have already progressed to a stage where vascular recovery alone is insufficient to prevent VF deterioration. This could explain why PPS VD recovery alone was associated with poorer outcomes in certain cases, as the damage may already be irreversible due to advanced LC deformation or ischemic insults.

In addition, as in our previous study, glaucoma progression was less in the laminar VD recovery group. When the IOP increases, the strain is mainly concentrated on the LC, and the capillaries between the crushed LC pores are compressed, resulting in VD reduction. If this progresses over a long period, extracellular matrix (ECM) remodeling of the LC occurs and VD recovery does not occur, even if the IOP decreases after glaucoma surgery. It can be determined indirectly through the recovery of the VD of the LC that remodeling has not progressed significantly. Therefore, the prognosis after glaucoma surgery is better in patients with less remodeling. Interestingly, the PPS VD significantly recovered even though in some eyes the laminar VD did not recover after glaucoma surgery. This suggests that ECM remodeling has not yet progressed in the case of PPS and is thought to be an indirect result showing that the structural change of the LC precedes that of the sclera in the progression of glaucoma. When the PPS VD recovered after surgery, the VF test showed worsening by 50%, which was different from the case of laminar VD recovery. First, it is highly likely that the deformation of the LC is already in progress when the PPS VD decreases; therefore, the damage to the axons passing through the LC continues even when IOP is normalized after glaucoma surgery. Second, the decrease in PPS VD indicates that the SPCAs were compressed, and there is a possibility that short-term ischemia was additionally applied to the LC during the period of elevated IOP, resulting in irreversible axonal damage. Therefore, despite the recovery of laminar VD in the case presented in Fig. 3, it is believed that glaucoma progressed rapidly.

In the multivariate analysis, PPS VD showed a significant correlation with laminar VD and significantly lower values with an increasing degree of myopia. This suggests that less deformation in the lamina corresponds to less deformation in the sclera, whereas more severe myopia results in increased strain on the peripapillary area due to high IOP. As the distance between the peripapillary arterial circle of Zinn-Haller, the primary arterial source for blood supply to the LC, and the optic disc border is significantly greater in highly myopic eyes^27^, ischemic damage to the LC due to lower PPS VD in myopia may be relevant to understanding why highly myopic eyes have increased susceptibility to glaucoma. A previous study indicated that in cases of high myopia, the improvement in the rate of VF deterioration after trabeculectomy is less significant than that in non-myopic cases^28^. Our previous research also observed that myopic eyes with large PPA tend to show greater central VF progression^29^. These results seem to be related to the PPS VD that we investigated in this study.

Our study has several limitations. The follow-up period was six months, during which we assessed VD values only at one month post-surgery. This decision stemmed from our previous findings indicating no significant difference in VD measurements between one- and three-months’ post-surgery^13^. However, it is possible that some patients experienced delayed VD responses to IOP reduction, potentially affecting our results. In this study, we specifically aimed to examine the short-term impact of VD changes on retinal ganglion cell function after glaucoma surgery. Longer-term observation may be complicated by disease progression or other risk factors. Those patients who exhibited delayed VD responses after one month post-surgery were categorized into the progression group. This suggests that patients with no or delayed responses tended to experience VF progression after glaucoma surgery. While promising, OCT-A imaging has limitations, particularly in analyzing segmented layers, especially deeper ones, owing to vascular shadowing or projection artifacts. While our OCT-A segmentation approach reflects current clinical standards, we acknowledge that further refinements in image processing software and artifact suppression algorithms may allow for more detailed and reliable analysis in future studies. Additionally, VD measurements may not fully capture the anatomical vessels. In our study, we considered deep VD in the laminar area to reflect the capillaries at the LC level. However, we aimed to mitigate the limitations of OCT-A visualization by evaluating the VD changes in the same area for each patient.

Another important limitation relates to the axial length of the eyes included in this study. The mean axial length of the study population was relatively long (25.45 ± 1.89 mm), which reflects a significant proportion of myopic eyes. Myopia is known to influence ocular microvasculature characteristics and scleral structure, which can affect OCT-A measurements and their interpretation. This factor is crucial when comparing our results to data from the general population or other studies that may include a more diverse range of axial lengths. The potential impact of myopia, including changes in scleral biomechanics and vascular architecture, should be considered when generalizing the findings of this study. Future studies may benefit from including a broader range of axial lengths to better contextualize the results and to assess the differential impact of myopia on VD changes and glaucoma progression.

In conclusion, we observed changes in the VD within the deep vascular layer of the ONH by dividing the region into the LC and PPS area up to one month after glaucoma surgery. The observation of laminar VD recovery after glaucoma surgery can be considered a very good indicator of the prognosis of glaucoma. The application of OCTA to assess microcirculation changes at the LC level holds promise for providing more accurate prognoses for patients with glaucoma undergoing surgery. In this study, the LC and PPS reacted differently depending on individual characteristics when IOP increased, and there was also a temporal precedence relationship of the change.

## Methods

### Subjects

We prospectively enrolled individuals diagnosed with primary open-angle glaucoma (POAG) who were scheduled for glaucoma surgical intervention at Seoul St. Mary’s Hospital between March 2017 and May 2020 because of uncontrolled elevated IOP. Ethical clearance for this study was granted by the Institutional Review Board of Seoul St. Mary’s Hospital, and the study complied with the ethical guidelines outlined in the Declaration of Helsinki. All eligible patients willing to participate were included successively, with each patient providing written informed consent.

All patients with POAG included in the study underwent comprehensive eye assessments. This included a review of their medical history, determination of their best-corrected visual acuity, assessment of refraction, examination with a slit-lamp biomicroscope, gonioscopy, Goldmann applanation tonometry, measurement of central corneal thickness using ultrasound pachymetry (Tomey Corp., Nagoya, Japan), determination of axial length through ocular biometry (IOL Master; Carl Zeiss Meditec, Dublin, CA, USA), a thorough examination of the optic disc using dilated stereoscopy, capturing red-free fundus images (Canon, Tokyo, Japan), employing Cirrus optical coherence tomography (OCT; Carl Zeiss Meditec), conducting Humphrey VF examination with the Swedish interactive threshold Standard 24–2 algorithm (Carl Zeiss Meditec), and performing OCT angiography (OCT-A) imaging (DRI OCT Triton; Topcon, Tokyo, Japan).

POAG diagnosis required a glaucomatous optic disc, indicated by either diffuse or localized rim thinning, a rim notch, or a vertical cup-to-disc ratio of ≥ 0.2 compared to the fellow eye. Additionally, a consistent VF finding of ≥ 3 non-edge points with < 5% probability, including one point < 1%, a PSD with a P value < 5%, or abnormal glaucoma hemifield test results on two VF examinations confirmed by specialists (H.Y.P. and C.K.P.). Finally, open-angle gonioscopy was performed.

The exclusion criteria encompassed several factors: best-corrected visual acuity worse than 20/40, spherical refraction beyond < − 8.0 diopters (D) or >  + 3.0 D, cylinder correction exceeding < − 3.0 D or >  + 3.0 D, medical history of retinal disease, eye trauma, or surgeries excluding uncomplicated cataract surgery, any optic nerve ailments except glaucoma, and systemic or neurological conditions potentially influencing the VF. In cases in which both eyes met the criteria, one eye was arbitrarily selected for examination.

The indications for glaucoma surgery were determined by assessing the progression of glaucomatous damage, whether in the VF, optic disc, or both, along with elevated IOP despite maximum tolerated medical therapy. All ocular hypotensive medications were continued until surgery. Postoperative complications including hypotony, shallow anterior chamber, choroidal detachment, and hypertensive phase were meticulously documented. Hypotony was defined as IOP < 6 mmHg within the first month post-surgery, while the hypertensive phase was characterized by a steady rise in IOP secondary to bleb encapsulation depending on whether the device used contained a valved mechanism^30^, and was defined as an IOP > 21 mmHg during the initial three-month postoperative period. Patients with hypotony, maculopathy, or disc edema were excluded from the study. Additionally, cataract progression, postoperative complications necessitating further intervention, and the requirement for a second procedure or surgery were considered as exclusion criteria.

### Optical coherence tomography angiography and determination of microvascular changes

The IOP and OCT-A images of the ONH were assessed one day prior to surgery and one month postoperatively. In our previous study, a gradual increase in the VD was observed until one month postoperatively, with minimal changes thereafter. Therefore, we chose one month after the procedure to conduct imaging^31^.

Images of the ONH region were captured using a commercially available swept-source OCT-A device (DRI OCT Triton; Topcon Corp.). This device utilized a central wavelength of 1050 nm, with an acquisition speed of 100,000 A-scans per second and axial and transversal resolutions of 7 and 20 µm, respectively. Scans were performed on cubes measuring 4.5 × 4.5 mm^2^, each containing 320 clusters of four repeated B-scans centered on the optic disc. To minimize motion artifacts, the device has an active eye tracker that monitors eye movements during image acquisition. Analysis was conducted on clear images with quality scores exceeding 30 to ensure minimal blurring due to motion or blinking. Subsequently, the acquired images were retrieved from the OCT-A device and imported into ImageJ software, a publicly available tool provided by the National Institutes of Health, Bethesda, MD, USA, for further processing and analysis.

VD measurements were made in the intradisc area and within the region of β-zone peripapillary atrophy (PPA). Details are in our previous studies^31–33^. In the area around the optic disc, we used automated software to segment different layers. Specifically, we focused on capturing the deep microvasculature surrounding the optic disc by delineating boundaries from 130 µm below the inner limiting membrane (ILM) to 390 µm below the basement membrane. This includes layers such as the inner nuclear layer (INL), outer plexiform layer, outer nuclear layer, and choroid. To quantify the vessel density (VD) within certain regions, we outlined the optic disc (Fig. 1A, yellow dotted line) and β-zone PPA (Fig. 1A, green dashed line) from accompanying photographs and overlaid them onto OCT-A images. VD within these regions was then measured, with the density of the region of β-zone PPA only calculated for eyes exhibiting this condition. In this study, the measurements of the VD of the deep layer of the optic disc area was named the laminar VD and that of the PPA area was named the PPS VD. We utilized ImageJ software to create binary images and determine the VD, with vessels represented by white pixels and the background by black pixels. Two observers (H.Y.P. and Y.J.) who were blinded to the clinical data independently measured these parameters on the OCT-A images, and their averaged measurements were used for analysis.

Significant changes in VD parameters were those surpassing the 95% Bland–Altman limits of agreement. Patients were divided into two groups, with and without VD changes in both the laminar and PPS VDs, according to the 95% limits.

### Determination of VF progression

Patients included in the analysis underwent two reliable VF tests conducted within a month before surgery and reliable follow-up VF tests at one-, three-, and six-months post-surgery (totaling five tests). The reliability criteria for the VF tests were fixation losses < 20%, false-positive responses < 15%, and false-negative responses < 15%. The same standards were applied to the baseline VF tests both before and after surgery. VF progression was determined using a linear regression analysis of the mean deviation (MD) values derived from five VF tests. MD progression rate was expressed as decibels (dB) change per year, with an MD slope <  − 1.0 dB/y indicating VF progression.

### Statistical analysis

VD assessment of 30 randomly chosen eyes was used to determine the interobserver reproducibility between the two observers (H.Y.P. and Y.J.) by computing intraclass correlation coefficients (ICCs) and their confidence intervals (CIs). Continuous and categorical variables were compared using Student’s t-test and the χ^2^ test, respectively. Univariate and multivariate linear regression analyses identified factors associated with VD parameters, with preoperative PPS VD, PPS VD changes, and preoperative laminar VD (Supplementary Table 1), laminar VD changes as the dependent variables and various independent variables including demographic data, ocular characteristics, and IOP and OCT parameters. Independent variables with P < 0.2 in the univariate analysis were included in the multivariate analysis. Statistical significance was set at P < 0.05. All analyses were conducted using SPSS software (version 16.0; SPSS Inc., Chicago, IL, USA).

## Supplementary Information

Supplementary Table 1.

## Data Availability

The datasets used and/or analyzed during the current study available from the corresponding author on reasonable request.
