# Supplementary material for: Microvasculature recovery in lamina cribrosa and peripapillary sclera after glaucoma surgery and its impact on visual field progression
Source: Sci Rep. 2025 Jul 11;15:25011. doi: 10.1038/s41598-025-08205-w (PMC12254377; doi:10.1038/s41598-025-08205-w)
Supplement: Supplementary file 1 — Supplementary Table 1. [file 41598_2025_8205_MOESM1_ESM.docx]

**Supplemental table 1. Factors associated with the preoperative laminar VD in glaucoma patients who undergone glaucoma surgery.**

| Variables | Univariate | | | | Multivariate | |
| --- | --- | --- | --- | --- | --- | --- |
|  | **Beta** | **95% CI** | ***P* Value** | **Beta** | **95% CI** | ***P* Value** |
| Age, per 1 y older  Axial length, per 1 mm larger  Central corneal thickness, per 1 μm thicker  Preoperative average pRNFL thickness, per 1 μm thicker  Preoperative average mGC/IPL thickness, per 1 μm thicker  Preoperative MD of VF, per 1 dB higher  Preoperative PSD of VF, per 1 dB higher  Preoperative IOP, per 1 mmHg higher  Postoperative IOP, per 1 mmHg higher  Preoperative PPS VD, per 1% higher | 7.670  -0.421  -0.049  -0.117  0.096  -0.038  0.138  0.113  0.391  0.372 | 0.834 to 14.507  -2.263 to 1.422  -0.121 to 0.023  -0.419 to 0.184  -0.324 to 0.516  -0.322 to 0.245  -0.322 to 0.598  -0.273 to 0.500  -0.150 to 0.933  0.015 to 0.729 | **0.029**  0.647  0.178  0.435  0.647  0.787  0.548  0.556  0.152  **0.042** | 3.055  0.298 | -1.035 to 7.146  0.057 to 0.540 | 0.141  **0.016** |

CI, confidence interval; pRNFL, peripapillary retinal nerve fiber layer; mGC/IPL, macular ganglion cell-inner plexiform layer; IOP, intraocular pressure; VF, visual field; MD, mean deviation; PSD, pattern standard deviation; dB, decibel; PPS, peripapillary sclera; VD, vessel density.

Factors with *P* < 0.2 in univariate analysis were included in multivariate analysis.

Factors with statistical significance are shown in bold.
